# How I do it ─ superficial parasternal intercostal plane catheter insertion

**DOI:** 10.1016/j.xjtc.2024.12.008

**Published:** 2025-01-08

**Authors:** Juan F. Morales, Michael J. Ricci, Subodh Verma, Fallon Dennis, Kyle Chin, Nitish K. Dhingra, S. M. Ali Hassan, Fábio de Vasconcelos Papa, Kendra L. Derry, Adrian Quan, Hwee Teoh, C. David Mazer, Ahmad Alli

**Affiliations:** a Department of Anesthesia, St Michael's Hospital of Unity Health Toronto, Toronto, Ontario, Canada; b Department of Anesthesiology and Pain Medicine, University of Toronto, Toronto, Ontario, Canada; c Division Cardiac Surgery, St Michael's Hospital of Unity Health Toronto, Toronto, Ontario, Canada; d Department of Surgery, University of Toronto, Toronto, Ontario, Canada; e Department of Pharmacology and Toxicology, University of Toronto, Toronto, Ontario, Canada; f Division of Endocrinology and Metabolism, St Michael's Hospital of Unity Health Toronto, Toronto, Ontario, Canada; g Department of Physiology, University of Toronto, Toronto, Ontario, Canada

**Figure:**
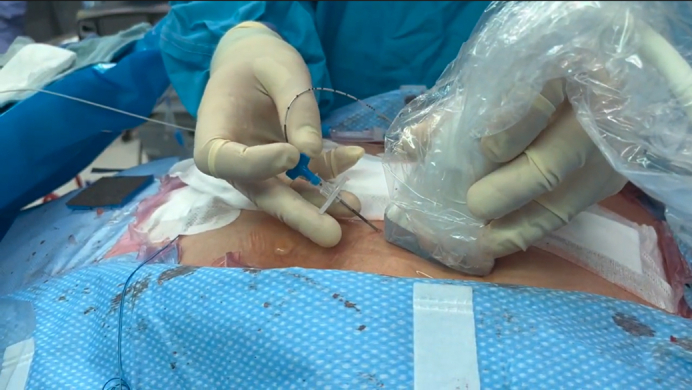
Insertion of the parasternal intercostal plane block catheter using ultrasound guidance.

**Central Message**

SPIP block for median sternotomy pain is practical and may represent a novel element of multimodal analgesia for enhanced recovery postcardiac surgery.

Anesthetic management for median sternotomy in cardiac surgery traditionally involves significant amounts of opioids. Reduction of perioperative opioid use is a priority for postcardiac surgery care, and the Enhanced Recovery After Surgery Society for Cardiac Surgery endorses regional and multimodal analgesic approaches.^1^^,^^2^

The overarching goal of postsurgical pain management is to achieve optimal analgesia with the least amount and severity of side effects. Regional anesthesia is key to opioid-sparing analgesia and can be a helpful component of multimodal analgesia. The combination of regional anesthesia with traditional pharmacological agents has been reported in some cases to improve resolution of chronic pain.^3^^,^^4^

The chest wall is innervated by the thoracic intercostal nerves that are ventral extensions of the thoracic spinal nerves; each intercostal nerve gives rise to a lateral cutaneous branch and terminates as the anterior cutaneous branch (Figure 1, *A*). The intercostal nerves and their branches may be anesthetized in a multiple spinal-segment fashion by injecting and allowing effective volumes of dilute local anesthetic (LA) to spread within discrete fascial planes.^5^

**Figure 1:**
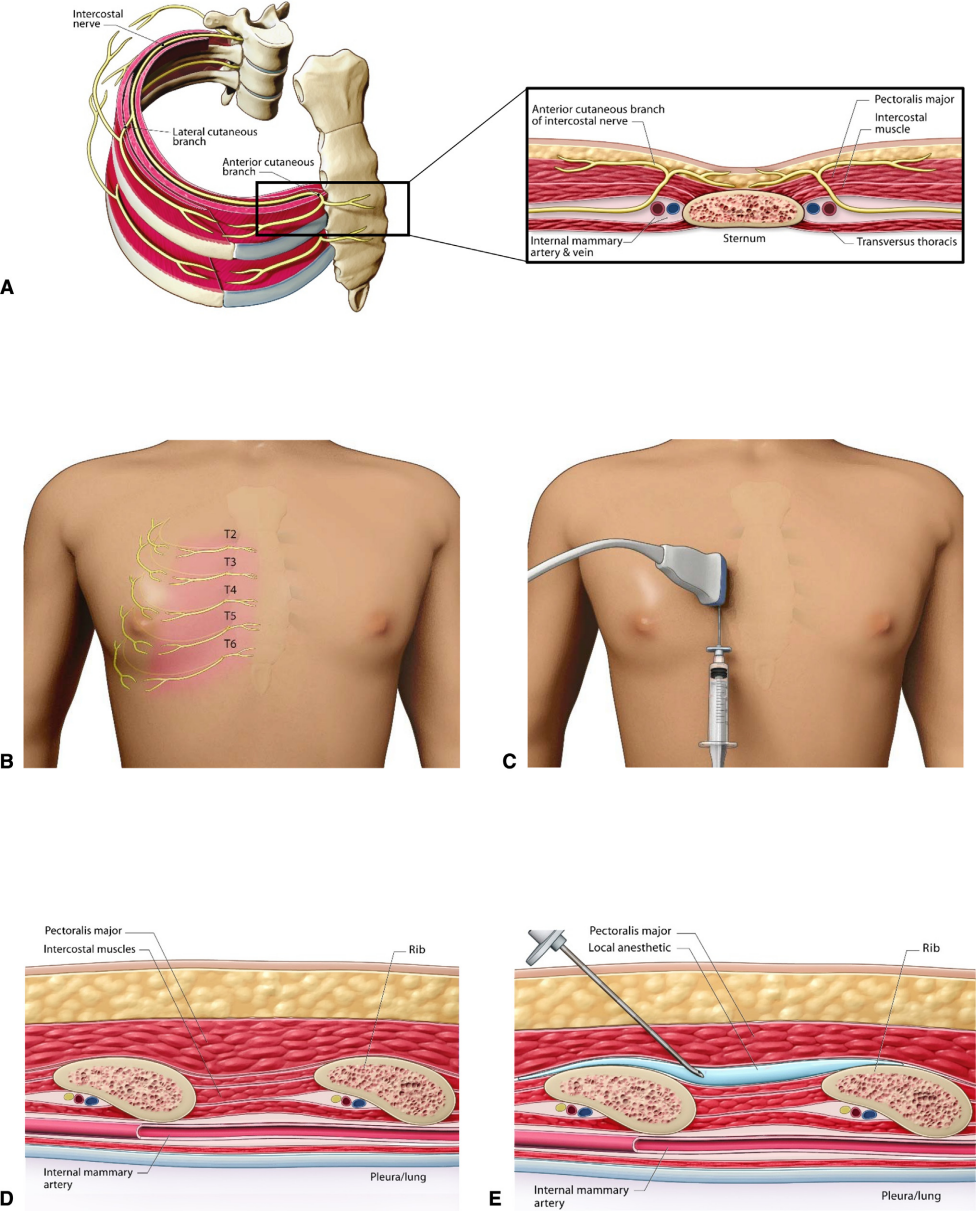
Ultrasound-guided SPIP block after median sternotomy in cardiac surgery. A, Anatomy of intercostal nerves and their branches. B, Sensory coverage of the anterior cutaneous branches of the right T2-T6 intercostal nerves. C, Positioning of ultrasound probe and injection site for right-sided SPIP block. D, Sagittal parasternal ultrasound landmarks for SPIP block. E, Posthydrodissection sagittal parasternal view of needle positioning for SPIP block. *SPIP*, Superficial parasternal intercostal plane.

The sternum is innervated by anterior cutaneous branches of the T2 to T6 intercostal nerves (Figure 1, *B*). Median sternotomy-related somatic pain may be managed by injecting LA into either the fascial plane between the pectoralis major muscle (PM) and intercostal muscle (IM) or between the IM and transversus thoracic muscle.^6^ These blocks have multiple names and are referred to as the superficial parasternal intercostal plane (SPIP) and deep parasternal intercostal plane blocks, respectively.

Our preferred technique for regional analgesia after median sternotomy is the SPIP block. Compared with deep parasternal intercostal plane blocks, SPIP blocks have more superficial targets and are less likely to cause accidental injury to the pleura and internal thoracic vessels.^7^

The paths of the lateral and anterior cutaneous branches of the intercostal nerves within and across definite continuous fascial planes can be monitored by ultrasound. We describe how we conduct SPIP blocks using programmed intermittent bolus to deliver adequate delivery of LA via a catheter placed under ultrasound guidance (Video 1).

• All procedures are conducted under strict aseptic conditions.
• After reversal of heparinization and closure of the sternum and skin, a linear, high-frequency ultrasound probe is placed in a sagittal parasternal orientation, approximately 2 to 3 cm from the lateral sternal border at the level of the fourth and fifth ribs (Figure 1, *C*).
• Ultrasound anatomic landmarks (eg, PM, IM, ribs, and pleura) are located (Figure 1, *D*). Vascular structures along the planned needle trajectory are detected by color Doppler.
• By using an “in-plane” ultrasound approach, a 16G or 17G Tuohy needle (or alternate catheter suitable nerve block needle) is inserted in the caudad–cephalad direction toward the middle to cephalad border of the fifth rib. The rib serves as “bony” protection from unintentional further needle advancement to deeper lying structures.
• Once contact with the rib is made, the needle tip position is optimized to enter the SPIP.
• Gentle hydrodissection is initiated with approximately 5 mL of inert injectate (normal saline or 5% dextrose). “Unzippering” of the PM and IM fascial layers and spread of the injectate along the cephalad–caudad axis are then confirmed. Special care is taken to ensure an intramuscular injection has not occurred because this can interfere with proper plane identification and catheter placement.
• The needle is advanced another 2 to 3 cm into the hydrodissected pocket. An additional 2 to 3 mL of injectate is introduced to promote further “unzippering” (Figures 1, *E* and 2, *A*).
• Once the needle tip is in the fascial plane and stabilized, the catheter is threaded and positioned 3 to 5 cm past the needle tip (Figure 2, *B*).

**Figure 2:**
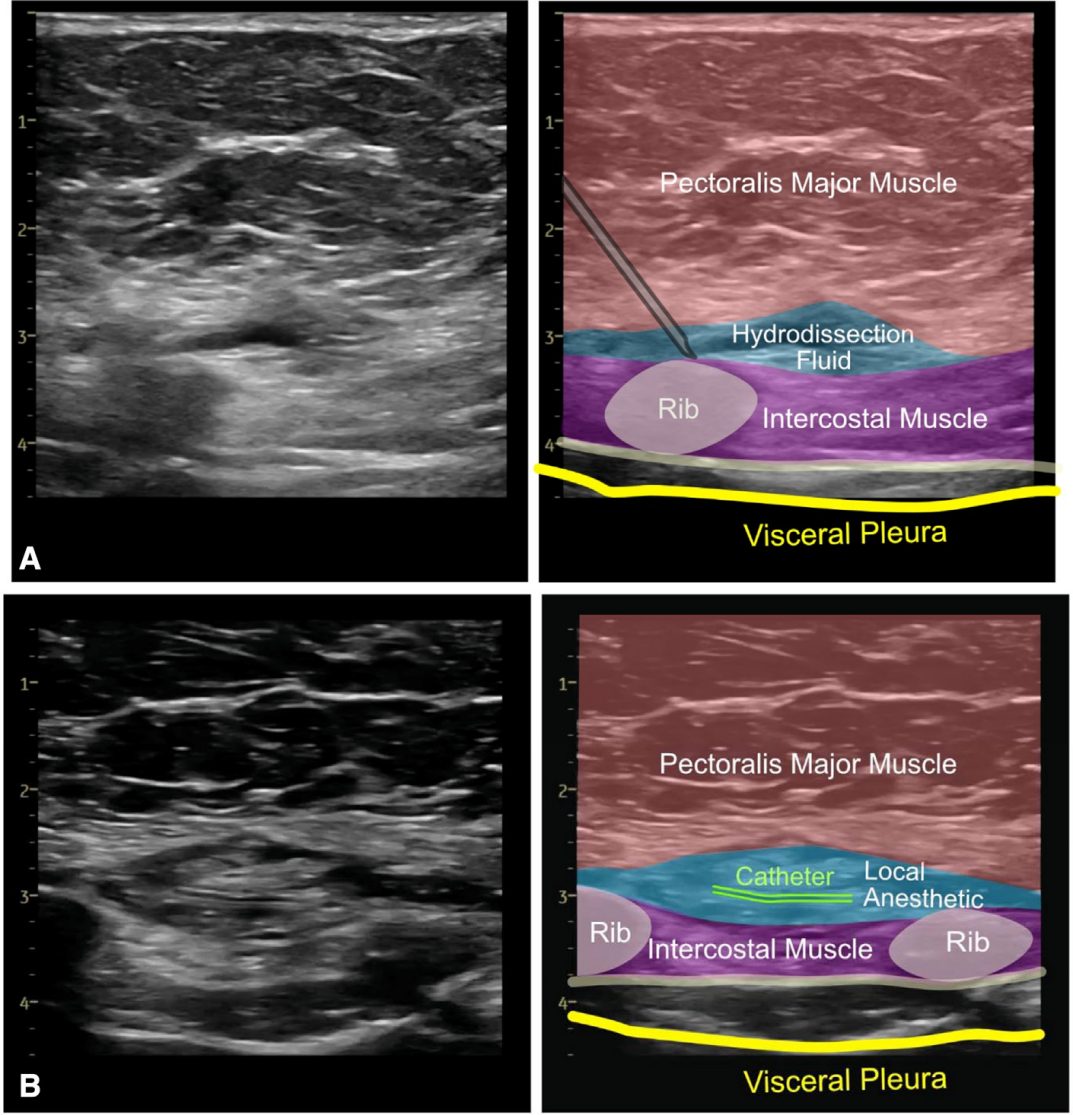
Linear transducer ultrasound images. A, Tuohy needle in the SPIP and spread of hydrodissection fluid. B, Positioning of nerve block catheter in the SPIP. *M*, Muscle; *SPIP*, superficial parasternal intercostal plane.
• The needle is then withdrawn, taking care not to dislocate the catheter.
• Placement of the catheter tip is visualized by ultrasound. If the catheter is confirmed to be correctly positioned by further “unzippering” of the fascial plane with 1 to 2 mL injectate, LA is injected via the catheter under ultrasound guidance. Regular aspiration attempts are made during the injection to rule out intravascular placement.
• The catheter is secured per standard practice with transparent dressings to ensure the entry site remains sterile. We use medical-grade cyanoacrylate glue (eg, Dermabond, Ethicon) to cover the entry points of the catheters and to avoid backflow or leakage of the LA.

Although small studies have shown a favorable safety profile, the large randomized controlled EPOCH CardioLink-10 trial is underway (ClinicalTrials.gov ID NCT06028126) and may offer insights into the potential of SPIP blocks as components of multimodal analgesia strategies for enhanced recovery postcardiac surgery, particularly when performed using the pragmatic technique described.

## Conflict of Interest Statement

S.V. holds a Tier 1 Canada Research Chair in Cardiovascular Surgery and reports receiving grants and/or research support and/or speaking honoraria from Amarin, Amgen, AstraZeneca, Bayer, Boehringer Ingelheim, Canadian Medical and Surgical Knowledge Translation Research Group, Eli Lilly, HLS Therapeutics, Humber River Health, Janssen, Merck, Novartis, Novo Nordisk, Pfizer, PhaseBio, S & L Solutions Event Management Inc, and Sanofi. He is the President of the Canadian Medical and Surgical Knowledge Translation Research Group, a federally incorporated not-for-profit physician organization. H.T. reports personal fees from the Canadian Medical and Surgical Knowledge Translation Research Group. C.D.M. is supported by a Merit Award from the University of Toronto Department of Anesthesiology and Pain Medicine; holds the Cara Phelan Chair in Critical Care at St Michael's Hospital-Unity Health Toronto; and has received has received Advisory Board honoraria/consulting fees from Amgen, Alexion, AstraZeneca, BioAge, Biotest, Boehringer Ingelheim, Cardior, CytoSorbents, ONA, PhaseBio, Sandoz, Trimedic Therapeutics, and Werfen, as well as Data Safety Monitoring Board stipends from Beth Israel Deaconess Medical Center, Cerus, and Takeda. A.A. reports consulting fees for lectures and advisory board involvement for Edwards Lifesciences. All other authors reported no conflicts of interest.

The *Journal* policy requires editors and reviewers to disclose conflicts of interest and to decline handling or reviewing manuscripts for which they may have a conflict of interest. The editors and reviewers of this article have no conflicts of interest.

## Supplementary Data

Video 1 Footage of the SPIP block being performed in real time with corresponding ultrasound loops. Video available at: https://www.jtcvs.org/article/S2666-2507(25)00001-X/fulltext.
