# Supplementary figures and images for: How I do it ─ superficial parasternal intercostal plane catheter insertion
Source: JTCVS Tech. 2025 Jan 8;30:107–10. doi: 10.1016/j.xjtc.2024.12.008 (PMC11998320; doi:10.1016/j.xjtc.2024.12.008)

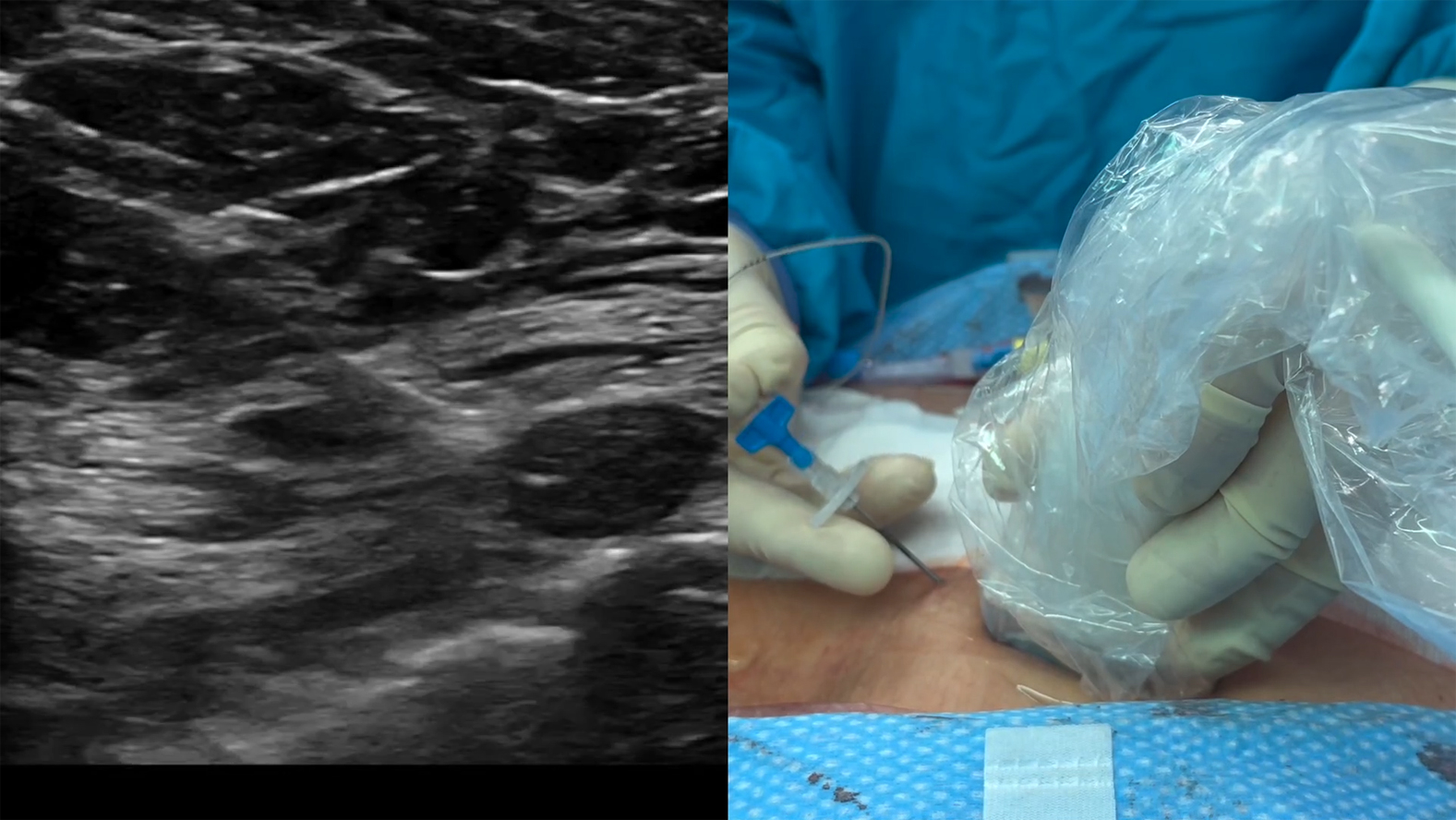

Supplement: Video 1 — Footage of the SPIP block being performed in real time with corresponding ultrasound loops. Video available at: https://www.jtcvs.org/article/S2666-2507(25)00001-X/fulltext. [file fx2.jpg]
